# Patterns of physical activity and sedentary behavior in a representative sample of a multi-ethnic South-East Asian population: a cross-sectional study

**DOI:** 10.1186/s12889-015-1668-7

**Published:** 2015-04-01

**Authors:** Aye Mya Win, Lim Wei Yen, Kristin HX Tan, Raymond Boon Tar Lim, Kee Seng Chia, Falk Mueller-Riemenschneider

**Affiliations:** Saw Swee Hock School of Public Health, National University of Singapore, Singapore, Singapore

**Keywords:** Physical activity, Exercise, Sedentary behavior

## Abstract

**Background:**

Few studies have investigated patterns of physical activity in a multi-ethnic Asian urban population. Even less is known about sedentary behaviors in these populations. The present study examined the prevalence of physical activity, exercise and sedentary behavior. In addition, it investigated socio-demographic correlates and the contribution of different domains towards overall physical activity.

**Methods:**

Data of 2319 participants from the population-based cross-sectional Singapore Health 2012 study were analyzed. Physical activity, exercise and sedentary behavior were assessed using the Global Physical Activity Questionnaires. A modified Cox regression model was used to estimate the relative prevalence rates (PR) for overall physical activity, leisure-time exercise and high level of sedentary behavior by socio-demographic factors.

**Results:**

Overall, 73.8% of participants met physical activity guidelines, 24.3% did regular leisure-time exercise and 37.0% reported high levels of sedentary behavior. Travel-related activities contributed about half of the total physical activity. There was a consistent association between age of participants with physical activity and exercise. Older participants were less likely to meet the guidelines (PR = 0.74, 95% C I = 0.61 – 0.91) than younger participants. The prevalence of regular exercise was lowest among 30 to 39 years aged participants (PR = 0.62, 95% CI = 0.45 – 0.86). Females exercised less regularly (PR = 0.63, 95% C I = 0.51 – 0.76) than males. Participants with higher education exercised regularly (PR = 2.08, 95% CI = 1.45 – 2.99) than participants with lower education. Employment status was consistently associated with exercise and high levels of sedentary behavior. Participants who were not in full-time employment exercised more regularly (PR = 1.45, 95% CI = 1.1 – 1.92) and were less likely to report high levels of sedentary behavior (PR = 0.65, 95% CI = 0.44 – 0.97) than those in full-time employment.

**Conclusions:**

Our population-based study suggests a need to encourage overall physical activity but, particularly regular leisure-time exercise, especially among middle-aged, females and those with lower levels of education and full-time employment. Strategies targeting workplaces may be important to reduce high levels of sedentary behavior.

## Background

The health benefits of physical activity have been widely documented. Regular moderate-intensity physical activity in adults can reduce the risk of hypertension, coronary heart disease, stroke, diabetes, breast and colon cancer, depression and risk of falls [1]. Physical inactivity causes more than 5.3 million deaths that occurred worldwide in 2008 [2]. WHO has stated that men are physically more active than women globally and the prevalence of insufficient physical activity, ie not meeting the WHO recommended physical activity guideline in South East Asia is lowest among all WHO regions with 15% for men and 19% for women [3]. Singapore is experiencing an increase in non-communicable diseases [4], for instance the prevalence of diabetes and obesity among 18 to 69 years Singaporeans has increased from 8.2% and 6.9% in 2004 to 11.3% and 10.8% in 2010 respectively [5]. Patterns of physical activity may play an important role in such developments and have been investigated in many countries [6-11]. However little is known about overall physical activity and exercise, but especially about domain-specific contributions towards physical activity, as well as their determining factors in the multi-ethnic Singaporean context. This specific information regarding the type of physical activity can be relevant, firstly because it has been suggested that the health benefits of certain types and intensities of activities might not be the same, and secondly, because public health implications and necessary health promotion strategies could differ depending on observed patterns [12-15].

More recently, sedentary behavior has been identified as an independent risk factor for many chronic diseases, such as diabetes, hypertension, heart disease, obesity, colon cancer and psychological distress [16-21]. Up to date systematic reviews and meta-analysis have confirmed these detrimental associations between sedentary behavior and health [22,23]. Sedentary behavior is defined as any waking behavior characterized by an energy expenditure not exceeding 1.5 metabolic equivalents (METs) while in a sitting or reclining posture [24]. Considering the negative impact of sedentary behavior on health, internationally there is a growing body of literature investigating sedentary behaviors in different population groups but cut-off points to define high levels of sedentary behavior have not been established [25-27]. A recent cohort study determined that prolonged sitting of more than 8 hours per day was significantly associated with higher all-cause mortality independent of physical activity [18]. Asian countries seem to have a particularly high level of sedentary behavior [28]. However, little is known about the situation in Singapore.

The overall goal of our study is to describe current patterns of physical activity, regular leisure-time exercise and high levels of sedentary behavior in a representative population-based study. This will help to guide the development of future health promotion strategies that aim to reduce the burden of overweight and obesity and chronic diseases. Specifically, we aim to determine the prevalence of sufficient physical activity, meeting exercise recommendations and engaging in high level of sedentary behavior. In addition we aim to investigate socio-demographic correlates and the contribution of different domains towards overall physical activity.

## Methods

### Study design and study population

**Figure 1:**
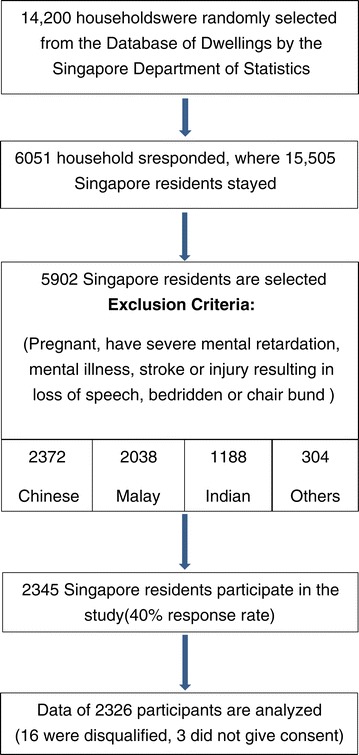
**Study flow chart.**

A cross-sectional survey was conducted between August 2012 and March 2013. Singapore is a tropical country with no seasons due to its close proximity to the Equator. The temperature is relatively stable throughout the months of the year and therefore, seasonal variation was not considered for this study [29]. A total of 14,200 households were selected randomly from the Database of Dwellings, a comprehensive database of all residential dwelling units in Singapore, maintained by the Singapore Department of Statistics [30]. Selected households were notified by post and this was followed up by house visits to enumerate all household members who met the inclusion criteria. Eligible households were defined as households where at least one person staying in the household met inclusion criteria. Inclusion criteria were Singaporeans or permanent residents who were born in the period of July 1933 to June 1994 and who stayed at least 4 days each week in the household and will be staying in the same household for the next 3 months or longer. Exclusion criteria were those who were pregnant, had severe mental retardation or mental illness (e.g. acute schizophrenia and dementia), had stroke or injury resulting in loss of speech and those who were bedridden or wheelchair bound. The excluded people especially those with functional limitation were considered unable to give informed consent to participate in the study and their lifestyle behavior may not be representative of their routine practice. From all the eligible households enumerated, a total of 5,902 Singapore residents from these households were selected to participate in the study with oversampling of Malays and Indians to improve the precision of prevalence estimates in these ethnic minority groups. (Figure 1) Selected individuals were invited to participate firstly by invitation letter, accompanied by participant information sheet and brochure and then followed by phone calls and household visits. Subsequently, an appointment for interview was set up for each participant. At the end of the interview, participants were invited for health examination. Ethics approval was obtained from the National University of Singapore Institutional Review Board and written informed consents were taken from the participants before starting the study.

### Assessment of physical activity and sedentary behavior

Physical activity was assessed using the Global Physical Activity Questionnaires (GPAQ) developed by the WHO. GPAQ is a valid and reliable tool for physical activity surveillance which has undergone rigorous evaluation and has been shown to be valid in different cultures [31]. Interviewers were trained to administer the GPAQ questionnaires to study participants. Participants were asked about the intensity and frequency of physical activity under 3 domains: work, travel to and from places, and recreation. According to the standardized approach of using GPAQ, vigorous- intensity activities are defined as activities that require a large amount of effort and causes rapid breathing and a substantial increase in heart rate. Moderate-intensity activities are defined as activities that require a moderate amount of effort and noticeably accelerate the heart rate.

Sedentary behavior was also assessed using GPAQ questionnaire. Participants were asked about time spent sitting or reclining on a typical day that includes sitting or reclining at work, at home, getting to and from places or with friends.

### Outcome variables

The outcome variables are defined as below.

### Meeting physical activity guidelines

Physical activity was assessed by using the cut-offs in the GPAQ analysis guide [32]. “Meeting physical activity guidelines or sufficiently active” is defined as doing at least

- 30 minutes of moderate – intensity activity or walking per day on at least 5 days in a typical week; or
- 20 minutes of vigorous – intensity activity per day on at least 3 days in a typical week; or
- 5 days of any combination of walking and moderate – or vigorous – intensity activities achieving a minimum of at least 600 MET – minutes per week.

MET (Metabolic equivalents) is the ratio of a person’s working metabolic rate relative to the resting metabolic rate. One MET is defined as the energy cost of sitting quietly, and it is equivalent to a caloric consumption of 1 kcal/kg/hour. MET values were calculated by multiplying weekly vigorous-intensity activity in minutes by 8 and weekly moderate-intensity activity in minutes by 4 [32]. The same cut-off value of meeting physical activity guideline or sufficiently active was commonly considered as sufficient physical activity in previous studies [33-35].

### Regular leisure-time exercise

Regular exercise was assessed by adapting the classification from the American College of Sports Medicine released in 2011 using the responses from GPAQ recreation domain. Regular exercise is defined as engaging in

- Moderate-intensity sports, fitness or recreational activities that cause a small increase in breathing or heart rate such as brisk walking, cycling, swimming, volleyball for ≥ 30 minutes per day on ≥ 5 days a week; or
- Vigorous-intensity sports, fitness or recreational activities that cause large increases in breathing or heart rate such as running, football for ≥ 20 minutes per day on ≥ 3 days a week; or
- A combination of moderate- and vigorous- intensity exercise to achieve a total energy of ≥ 500 – 1000 MET minutes per week [36]. The conceptually same cut-off value for assessing leisure-time exercise was used in previous studies [37,38].

### Contribution of activity types to overall physical activities

To determine the contribution of work, travel and recreational domains to overall level of physical activity, MET minutes per week per individual were first calculated for each domain. Then, the percentages of contribution of each domain towards total physical activity were calculated and the mean percent contribution was assessed.

### Sedentary behavior

We dichotomized the study population into two groups based on sitting time on a typical day: those sitting at least 8 hours per day or high level of sedentary behavior, and those sitting less than 8 hours per day. This dichotomization is based on the finding from a previous study that reported a detrimental association between sitting more than 8 hours a day and all- cause mortality [18].

### Data processing and statistical analysis

Due to the study methodology, we were not able to collect detailed information from non-respondents. However, we applied sample weights to be representative of Singapore general population. Data were analyzed using SPSS 21 and STATA 11. Data cleaning on physical activity responses was done according to GPAQ data cleaning instructions [32]. The prevalence of meeting physical activity guideline, regular leisure-time exercise to improve and maintain physical fitness and health, sedentary behavior and contribution of activity types to overall physical activities were estimated after weighting the data. Subsequently, association of these behaviors and socio-demographic characteristics were estimated with chi-square test. We evaluated socio-demographic variables such as age, gender, and ethnicity (as listed in the National Registration Identity Card; ethnicity in these documents was defined by paternal ethnicity), and highest level of education attained, marital status, employment status and average household income as the independent variables. The categorization of all socio-demographic variables as used in the current study is shown in Table 1. Among all the variables, age is categorized into five groups from the original response. Race, highest level of education attained, marital status, employment status and monthly household income are categorized from the original answering formats.

**Table 1:** **Socio- demographic characteristics of study population**

	Males (n = 1128) %*	Females (n = 1191) %*	Overall,(n = 2319) %*
**Mean age**	43.2 ± 15.1 years	42.9 ± 15.2 years	43.0 ± 15.2 years
**Age group**			
18 - 29 years	270 (21.8%)	277 (22.1%)	547 (21.9%)
30 - 39 years	204 (21.1%)	219 (22.0%)	423 (21.6%)
40 - 49 years	254 (22.2%)	291 (22.0%)	545 (22.1%)
50 - 59 years	240 (19.5%)	239 (17.9%)	479 (18.7%)
60 - 79 years	160 (15.5%)	165 (16.0%)	325 (15.7%)
**Race/ethnicity**			
Chinese	526 (75.2%)	371 (75.7%)	797 (75.4%)
Malay	355 (12.3%)	451 (12.5%)	806 (12.4%)
Indian	284 (9.4%)	311 (8.5%)	595 (8.9%)
Others	63 (3.1%)	58 (3.4%)	121 (3.2%)
**Highest level of education attained**			
Secondary and below	276 (20.2%)	384 (24.6%)	660 (22.4%)
GCE O/N level	297 (19.9%)	329 (25.4%)	626 (22.7%)
GCE A/diploma/professional qualification	170 (17.2%)	150 (13.4%)	320 (15.3%)
Polytechnic, University and Above	383 (42.7%)	327 (36.6%)	710 (39.6%)
**Marital status**			
Not currently married	341 (30.6%)	424 (39.2%)	756 (35.0%)
Currently married	787 (69.4%)	766 (60.8%)	1553 (65.1%)
**Employment status**			
Full-time employment (work/Student/National Service)	998 (88.8%)	792 (71.8%)	1790 (80.3%)
Not full-time employment	128 (11.2%)	399 (28.2%)	527 (19.8%)
**Monthly household income over the last 12 months**			
Below S$ 2,000	203 (14.6%)	219 (16.8%)	422 (15.7%)
S$ 2,000 to 3,999	318 (27.5%)	348 (25.5%)	666 (26.5%)
S$ 4,000 to 5,999	235 (19.0%)	230 (20.8%)	465 (19.9%)
S$ 6,000 and above	280 (38.9%)	266 (36.9%)	546 (37.9%)

*The percentages are weighted percentage.

Finally, prevalence ratios adjusted for socio-demographic characteristics for each behavior were estimated using cox regression model. In our study, we used a modified cox regression model to better estimate the prevalence ratio of the outcomes, since odds ratios obtained in logistic regression can overestimate the prevalence rate ratio when the condition under study is highly prevalent [39,40].

### Sample weights

Sample weights were calculated for both household enumeration exercise and the study fieldwork. For the household enumeration exercise, sample weights (W_EE_) comprised weights for unequal probability of selection and non-response that were computed based on three attributes, namely the household ethnicity, dwelling type and region of dwelling.

For the study fieldwork, sample weights (W_SF_) comprised weights for unequal probability of selection and non-response that were computed based on four attributes — dwelling type, region of dwelling, ethnicity and age. Post-stratification weights (W_PS_) were computed based on the ethnicity, age and gender attributes. The overall sample weights was the product of W_EE_, W_SF_ and W_PS_.

## Results

Out of 5902 selected participants, a total of 2345 participated in the study with a 40% response rate. From these participants, 16 were disqualified and 3 did not give consent to use the data. The analysis was performed on data of the remaining 2326 participants. Out of 2326, there were 7 participants who did not answer the physical activity questionnaires and they were excluded from analysis.

The socio-demographic characteristics of 2319 study participants stratified by gender are shown in Table 1. The mean age of study participants was 43 ± 15.2 years with similar distribution of males and females among different age groups. Majority of participants were Chinese (75.4%), attained highest education of polytechnic, university and above (around 40%), currently married (65.1%), with full-time employment (around 80%) and had average monthly household income of S$ 6000 and above (around 40%). (Table 1)

### Prevalence of meeting physical activity guidelines overall and by socio-demographic characteristics

**Figure 2:**
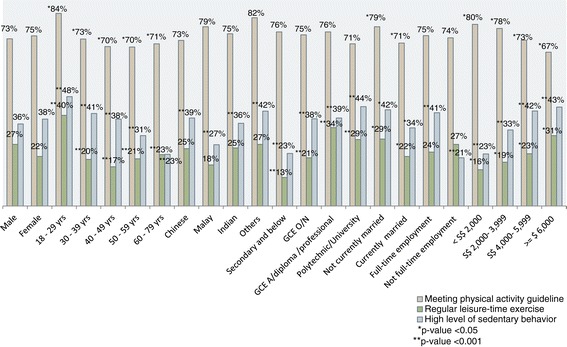
**Prevalence of physical activity, regular leisure-time exercise and high level of sedentary behavior by socio-demographic characteristics.** P-value indicates significant differences between variable categories within each socio-demographic characteristic. The percentages presented are weighted percentages.

Overall, the proportion of the study population that met physical activity guideline was 73.8%. The prevalence was highest among those aged 18 to 29 years (84.3%) and lowest among 40 to 59 years (around 70%). The prevalence was reported by a higher proportion of participants who were not currently married (78.5%) compared to currently married participants (71.3%). The prevalence was highest among participants with monthly household income less than S$ 2000 and seemed to decrease with increasing monthly household income (Figure 2).

### Prevalence of regular leisure-time exercise overall and by socio-demographic characteristics

Overall, the proportion of the study population who engaged in regular leisure-time exercise was 24.3%. The prevalence was highest among youngest participants aged 18 to 29 years (40%) and lowest among 30 to 49 years (17%). The prevalence was reported by a lower proportion of participants with secondary and below level of education (13%) compared to those with higher levels of education. The prevalence seemed to increase with increasing educational level. A higher proportion of participants who were not currently married exercised regularly than those who were currently married (29% vs 22%). The prevalence was highest among participants with monthly household income S$6000 and above (31%) (Figure 2).

### Prevalence of high level of sedentary behavior overall and by socio- demographic characteristics

The median sitting hours of participants on a typical day was 6 (IQR: 3, 8) hours. Around 37% of Singaporeans sat at least 8 hours per day (high level of sedentary behavior). The prevalence was highest among youngest participants aged 18 to 29 years (47.9%). The prevalence decreased with increasing age groups. Chinese participants had highest prevalence (38.5%) followed by Indians (36.2%) and Malays (26.8%). High level of sedentary behavior was reported by 44% of participants with polytechnic, university and above level of education. The prevalence increased with increasing educational level. A higher proportion of participants who were not currently married reported high level of sedentary behavior than among currently married participants (42.1% vs 34.2%). Participants who were in full-time employment were more likely to have high level of sedentary behavior (around 41%) than those who were not in full-time employment (21.1%). The prevalence of high level of sedentary behavior was highest among participants with monthly household income of S$ 6000 and above (43.3%), and this prevalence increased with increasing monthly household income (Figure 2).

### Domain specific contribution of activities towards overall physical activities

**Figure 3:**
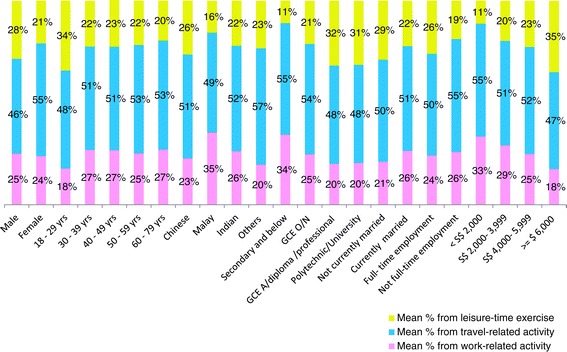
**Domain specific contribution of activities towards overall physical activities by socio-demographic characteristics.** There were 97 participants who did not contribute to any domains. These 97 participants were excluded from analysis on domain-specific contribution towards overall physical activity.

In total, the mean contribution of travel-related activity towards total physical activity was largest (50.9%), followed by leisure-time exercise (24.6%) and work-related activity (24.5%). The dominant pattern of travel-related activity was seen across different socio-economic groups (Figure 3).

### Association of meeting physical activity guidelines, regular leisure-time exercise and high level of sedentary behavior

Table 2 shows the adjusted prevalence ratios (PR) with 95% CI for meeting physical activity guidelines, doing regular leisure- time exercise and high sedentary behavior.

**Table 2:** **Association of meeting physical activity guidelines, regular leisure-time exercise and high level of sedentary behavior**

	**Meeting physical activity guidelines**			**Regular leisure-time exercise**			**High level of sedentary behavior**		
	**PR**	**95% CI**	**P value**	**PR**	**95% CI**	**P value**	**PR**	**95% CI**	**P value**
			**0.03**			**<0.001**			**<0.001**
**Age Group**									
18 - 29 years	1.00			1.00			1.00		
30 - 39 years	0.86	(0.75–0.98)	0.030	0.62	(0.45–0.86)	0	0.90	(0.67–1.20)	0.500
40 - 49 years	0.79	(0.68–0.90)	0	0.66	(0.48–0.91)	0.010	0.89	(0.66–1.20)	0.500
50 - 59 years	0.78	(0.66–0.93)	0.01	0.73	(0.52–1.01)	0.060	0.80	(0.54–1.20)	0.300
60 - 79 years	0.74	(0.61–0.91)	0	0.72	(0.48–1.10)	0.130	0.73	(0.46–1.13)	0.160
**Gender**									
Male	1.00						1		
Female	1.00	(0.92–1.09)	0.900	0.63	(0.51–0.76)	<0.001	1.11	(0.93–1.33)	0.250
**Race/ethnicity**									
Chinese	1.00			1.00			1.00		
Malay	1.03	(0.96–1.10)	0.400	0.06	(0.75–1.23)	0.760	0.83	(0.70–0.99)	0.040
Indian	1.03	(0.95–1.10)	0.500	1.09	(0.86–1.38)	0.500	1.00	(0.84–1.18)	1.000
Others	1.16	(1.02–1.32)	0.020	1.03	(0.68–1.58)	0.900	1.10	(0.84–1.42)	0.500
**Highest level of education attained**									
Secondary and below	1.00			1.00			1.00		
GCE O/N level	0.94	(0.83–1.05)	0.300	1.69	(1.22–2.33)	0	1.35	(0.99–1.84)	0.060
GCE A/diploma/professional qualification	0.97	(0.84–1.13)	0.700	2.08	(1.45–2.99)	<0.001	1.21	(0.83–1.76)	0.300
Polytechnic/University and Above	0.92	(0.81–1.05)	0.200	2.00	(1.40–2.84)	<0.001	1.30	(0.90–1.88)	0.160
**Marital status**									
Not currently married	1.00			1.00			1.00		
Currently married	1.03	(0.81–1.05)	0.200	0.86	(0.66–1.11)	0.240	0.93	(0.71–1.20)	0.600
**Employment status**									
Full-time employment (work/student/national service)	1.00			1.00			1.00		
Not full-time employment	1.03	(0.92–1.17)	0.600	1.45	(1.10–1.92)	0.01	0.65	(0.44–0.97)	0.030
**Monthly household income over the last 12 months**									
Below S$ 2,000	1.00			1.00			1.00		
S$ 2,000 to 3,999	0.96	(0.87–1.07)	0.500	0.94	(0.69–1.27)	0.680	1.29	(0.89–1.87)	0.180
S$ 4,000 to 5,999	0.90	(0.80–1.02)	0.100	1.08	(0.79–1.49)	0.630	1.46	(0.99–2.15)	0.060
S$ 6,000 and above	0.84	(0.74–0.97)	0.010	1.31	(0.95–1.81)	0.090	1.49	(0.99–2.24)	0.050

Age and monthly household income over the past 12 months of participants were significantly associated with meeting physical activity guidelines. The prevalence was lowest among oldest participants aged 60 to 79 years (PR = 0.74, 95% CI = 0.61 – 0.91) compared to youngest participants aged 18 to 29 years. The prevalence declined with increasing age groups. Participants with monthly household income $S 6000 and above were least likely to meet physical activity guidelines (PR = 0.84, 95% CI = 0.74 – 0.97). The prevalence declined with increasing income.

Age, gender, highest educational level attained and employment status were significantly associated with regular leisure- time exercise. The prevalence was lowest among participants aged 30 to 39 years (PR = 0.62, 95% CI = 0.45 – 0.86) compared to youngest participants aged 18 to 29 years. Females were less likely to exercise regularly (PR = 0.63, 95% CI = 0.51 – 0.76) than males. Participants with higher educational level were more likely to exercise regularly (PR = 2.08, 95% CI = 1.45 – 2.99) than participants with lower education level. The prevalence increased with increasing educational level. Participants who were not in full-time employment were more likely to exercise regularly (PR = 1.45, 95% CI = 1.1 – 1.92) than those who were in full-time employment.

Only employment status was significantly associated with high level of sedentary behavior. Participants who were not in full-time employment were less likely to sit at least 8 hours on a typical day (PR = 0.65, 95% CI = 0.44 – 0.97) compared to those who are in full-time employment (Table 2).

Prevalence ratios of outcomes adjusted for socio-demographic characteristics were estimated separately for males and females. However, the results did not change substantially and therefore, the results are not presented.

## Discussion

In this study, the prevalence of Singaporeans who met physical activity guidelines was 73.8%. In contrast, less than a quarter of Singaporeans did regular exercise during leisure time. Around half of the total physical activity was contributed by travel-related activities. The median sitting hours of participants on a typical day was 6 (IQR: 3, 8) and high levels of sedentary behavior were found in almost 40% of Singaporeans. We observed that participants who were older and with higher monthly household income were less likely to meet overall physical activity guidelines. However, middle-aged participants, females, participants with lower educational level and participants who were in full-time employment were less likely to exercise regularly. High levels of sedentary behavior were observed among participants who were in full-time employment.

The percentage of Singaporeans who met physical activity guideline from our study seemed somewhat higher compared to global estimates (69%) but lower than in Southeast Asia (83%) [41]. Within the Asia Pacific region, the prevalence of Singaporeans meeting physical activity guidelines was higher than in Malaysia (40%), Kiribati (50%) and Vietnam (58%) but lower than in India (84%), Indonesia (78%), Mongolia (89%) and Sri Lanka (85%) [42]. However, the variation in prevalence across countries may also depend on sampling methodology, culture and policy in place.

The prevalence of leisure-time exercise was substantially lower than in a study from Taiwan that used a similar methodology [37]. Comparisons with other countries are limited due to differences in assessment methodology [38,43,44].

In our study, there was a consistent association between age of participants with physical activity and regular leisure-time exercise. Younger participants were generally more active and exercised more than older participants. A similar trend of higher physical activity level among younger participants had previously been observed [34]. Surprisingly, middle aged participants (30 to 49 years) exercised least regularly, similar to the Taiwanese study [37]. Competing commitments in this age group due to career or family may explain these findings. Similar to our observation of low exercise levels in middle aged adults, an almost simultaneous increase in obesity in this age group has been reported, which may partly be explained by our findings [5,45]. Health promotion strategies targeting this middle-aged working population may therefore be important.

Our study shows considerable differences between the prevalence of Singaporeans who met physical activity guidelines and the prevalence of regular leisure-time exercise according to recommendations. This can be explained by the large contribution of travel-related activity towards overall physical activities. The contribution of travel-related activities towards total physical activities was frequently reported in developing countries [11,46], but less often in industrialized countries [47]. Although most recommendations nowadays advise a certain level of total physical activity others, such as from the American College of Sports Medicine are more focused towards leisure time exercise [36,48]. The low level of leisure-time exercise as compared to travel-related activity and activities from other domains may have important public health implications. Firstly, travel-related activities may be of lower intensity and it has been suggested that their health effects might be smaller than those of exercise [12,49]. Secondly, purposeful exercise may generate effects, such as improved fitness, flexibility, strength or even quality of life, which go beyond simply improving overall health [12,13,15,36]. Thirdly, low levels of regular exercise as compared to relatively high levels of total physical activity in Singapore, could have implications for the choice of appropriate health promotion activities that aim to promote physical activity at the population level. For instance, the dominant pattern of travel-related activity across different socio-demographic characteristics may reflect a supportive transport infrastructure or public transport system, while on the other hand other strategies, such as creating awareness of exercise facilities, work-place physical activity promotion activities, a more supportive workplace infrastructure or workplace and family policies may need to be looked into with regard to their supportiveness of leisure time activities.

The current study finds some inconsistencies. For example, participants with lower education level and lower household income seemed more likely to meet physical activity guidelines than those with higher education and higher income group. However, the reverse pattern was seen for exercise. Participants with lower education and lower income may work in the employment sectors where more work-related activity is needed, which may contribute to their overall physical activity. On the other hand, higher educational levels and higher income employments may be associated with less occupational activity but these individuals may deliberately engage in more leisure-time exercise. Although statistically not significant, married individuals seemed to do less exercise and be less sedentary than those not married. A possible explanation is that due to children or other family commitments, they engage in more unintentional physical activity but at the same time have less time for structured exercise.

High levels of sitting time were previously compared across 20 countries using a cut-off of 9 hours. To compare with these previous estimates, we additionally assessed sedentary behavior using identical same cut-off, which resulted in a prevalence of 25%. This is similar to the prevalence in Hong Kong and higher than most countries including US and Australia [28]. High levels of sedentary behavior were more likely in full-time working adults and in those with higher educational level, which may be explained by the long working hours in Singapore and their employment in predominantly sedentary white collar occupations. Our study suggests that younger participants seemed to sit more than their older counterparts, which is different from previous findings from other countries [28]. This finding may reflect the educational profile of Singaporeans where young/middle aged adults tend to attain higher education levels [50], which may result in more sedentary occupations. We also note that those less active were also more likely to engage in high levels of sedentary behavior (data not shown), which may lead to accumulating detrimental health effects due to those independent risk factors. Our findings of high levels of sedentary behavior and little leisure-time exercise, particularly in middle-aged working adults suggest strategies targeting this population and workplaces could be a key public health target in this multi-ethnic Asian population.

By using the GPAQ, we used a validated questionnaire that covers different domains and intensity categories of physical activity, and offers validated cut-offs for the classification of individuals. However, assessment of sitting time by GPAQ is restricted to only one question without further differentiation by domains [31,51]. Future population-based studies should aim to determine physical activity and sedentary behavior more reliably using objective approaches to reduce recall bias. Another limitation is the relatively low overall response rate. This may have distorted our prevalence estimates but should not affect the observed associations.

## Conclusion

This study describes the prevalence of physical activity, exercise and sedentary behavior in a multi-ethnic Asian population. More than 70% of Singaporeans met physical activity guidelines, which is higher than in many other countries. In contrast, regular leisure- time exercise was low compared to other countries and about half of total physical activity was contributed by travel-related activities. Furthermore, almost 40% of Singaporeans spent at least 8 hours per day sitting, which has been identified as an independent risk factor for premature mortality.

Middle-aged adults seemed to engage in particularly low levels of physical activity and exercise. This is paralleled by a substantial increase in obesity prevalence in this age group. Based on our findings, public health intervention strategies that target middle- aged adults seem to be particularly important to counter increasing trends in obesity during this time of life. Work place strategies to reduce sedentary behaviors could be another promising approach to reduce the high levels of sedentary behavior and to improve energy balance in this population. Future studies should aim to explain the discrepancy between overall physical activity levels and low levels of regular leisure-time exercise. To this end, more objective assessments of physical activity and sedentary behavior will be important to assess these behaviors more reliably. Finally efforts should be made to develop effective and feasible public health interventions aimed at relevant population groups and settings.

## Abbreviations

WHO: World Health Organization
GPAQ: Global physical activity questionnaires
METs: Metabolic equivalents
TV: Television
GCE: General Certificate of education
IPAQ: International physical activity questionnaires
IQR: Interquartile range
PR: Prevalence ratio
CI: Confidence interval
